# Inhibitory effect of HGF on invasiveness of aggressive MDA-MB231 breast carcinoma cells, and role of HDACs

**DOI:** 10.1038/sj.bjc.6604726

**Published:** 2008-10-21

**Authors:** E Ridolfi, E Matteucci, P Maroni, M A Desiderio

**Affiliations:** 1 Institute of General Pathology, University of Milan, Milan, Italy; 2 Istituto Ortopedico Galeazzi, IRCCS, Milan, Italy

**Keywords:** hepatocyte growth factor, breast cancer, CXCR4, signalling pathways, HDAC inhibitors, trichostatin A

## Abstract

Hepatocyte growth factor (HGF), through Met receptor binding, fulfils numerous functions in invasive tumour growth (survival/proliferation, motility, apoptosis), but epigenetic control of gene expression in this process is poorly understood. In HGF-treated breast cancer cells we studied (a) the chemoinvasion towards CXCL12 (ligand of the chemokine-receptor CXCR4) and (b) the mechanistic basis, that is, the transduction pathways that regulate CXCR4-mediated invasion, and the role played by histone deacetylases (HDACs) after blockade with trichostatin A (TSA). In highly invasive and metastatic MDA-MB231 cells HGF had a dual inhibitory effect, reducing spontaneous migration and specific chemoinvasion towards CXCL12, the latter by decreasing CXCR4 transactivation and protein level. After HGF the levels of phosphorylated (therefore active) c-Src and Akt persistently increased, indicating a role of these signal transducers in the HGF-dependent cellular and molecular effects. c-Src wild-type expression vector (Srcwt) increased active c-Src and mimicked the HGF-dependent inhibition of CXCR4 transactivation. Our findings indicate that HDACs participated in the HGF-inhibitory effects. In fact, blockade of HDACs hindered the HGF- and Srcwt-dependent reductions of CXCR4 transactivation and invasiveness, while inhibition of endogenous c-Src was additive with HGF, further reducing specific chemoinvasion. In conclusion, in MDA-MB231 cells HDAC blockade with TSA partly counteracted the HGF-dependent effects through molecular events that included enhancement of the expression of the genes for invasiveness Met and CXCR4 (depending on serum conditions), reduction of endogenous phospho-c-Src/c-Src and phosphoAkt/Akt ratios and triggering of apoptosis. The potential therapeutic use of TSA should take into account the variable aggressiveness of breast carcinoma cells and microenvironment signals such as HGF at the secondary growth site of the tumour. It was interesting that HGF reduced motility and CXCR4 functionality only of MDA-MB231 cells, and not of low-invasive MCF-7 cells, suggesting a mechanism implicated in metastatic cell homing.

Hepatocyte growth factor (HGF) is a multifunctional cytokine produced by cells of the supportive tumour microenvironment, and a central regulator of the invasive/metastatic phenotype of neoplastic cells (Matsumoto and Nakamura, 2006; Mazzone and Comoglio, 2006; Desiderio, 2007). Through Met receptor binding, HGF aberrantly activates the motility/chemoinvasion, proliferation/survival and apoptosis that characterize the epithelial-mesenchymal transition of aggressive carcinomas. The HGF/Met couple controls the expression of a panel of genes important for these processes, including HIF-1*α* (the inducible subunit of HIF-1 transcription factor), members of the plasminogen activation system and the C-X-C motif receptor 4 (CXCR4) (Desiderio, 2007; Gordan and Simon, 2007).

Chemokine receptor CXCR4 is involved only in some partially clarified steps of carcinoma (breast, prostate) metastatic process (Balkwill, 2004; Darash-Yahana *et al*, 2004), and silencing CXCR4 blocks breast cancer metastasis (Liang *et al*, 2005). Organs that are the first destination of breast cancer metastases have high levels of CXCL12, the CXCR4-specific ligand (Balkwill, 2004). The expression of CXCR4 is lower in lymph node metastases than in primary breast cancer (Shim *et al*, 2006). In low (MCF-7) and highly (MDA-MB231) invasive breast carcinoma cells (Guo *et al*, 2002; Yang *et al*, 2007), HGF respectively raises and lowers the expression of CXCR4 (Matteucci *et al*, 2005).

The MDA-MB231 cell line is derived from pleural effusion fluid and is used as a model for metastatic cell migration. In view of its gene expression profile and strong invasive behaviour *in vitro*, this is a highly dedifferentiated mesenchymal-like cell line, which has lost most epithelial markers and character, and expresses mst1 consistent with the strong metastatic behaviour (Betapudi *et al*, 2006). In MDA-MB231 cells, histone deacetylase 3 (HDAC3) and phospho-c-Src colocalize at the cell membrane level early after HGF exposure (Matteucci *et al*, 2007), but the possible correlation with the reduction of CXCR4 level has never been established and the functional significance for invasiveness has not been explained.

Histone deacetylases participate in the aberrant epigenetic control of gene expression in tumours through deacetylation of histones and numerous transcription factors, as well as cytosolic proteins, regulating cell proliferation, cell cycle and apoptosis (Carey and La Thangue, 2006; Ropero and Esteller, 2007). We set out to verify whether in HGF-exposed breast carcinoma cells, HDACs were important for invasive growth by regulating gene expression, motility and apoptosis, depending on the tumour aggressiveness.

This paper focuses on chemoinvasion towards CXCL12 in invasive/metastatic MDA-MB231 breast carcinoma cells after HGF treatment, looking into the mechanistic basis, which might differ from MCF-7 cells. We examined the signal-transduction pathways that might possibly regulate the changes of CXCR4 transactivation and protein levels, and invasiveness after HGF, and the role of the HDACs. Trichostatin A (TSA), a hydroxamic-based compound that inhibits Class I and II HDACs (Cameron *et al*, 1999), was used to assess the involvement of HDACs. Histone deacetylases are considered important cancer targets, and HDAC inhibitors hold out promise as a new class of anticancer drugs (Carey and La Thangue, 2006). Trichostatin A shows antitumour activity against breast cancer *in vivo* and *in vitro* (Yoshida *et al*, 1990; Vigushin *et al*, 2001; Blagosklonny *et al*, 2005).

The tyrosine kinase c-Src and the downstream effectors ERK1/2 and Akt lie along the HGF/Met signal-transduction pathway (Mazzone and Comoglio, 2006), and might be involved in the regulation of NF-*κ*B transcription factor (Huang *et al*, 2003a; deGraffenried *et al*, 2004; Arthur, 2008). Nuclear factor-*κ*B leads to an increase in the invasive capacity of breast and ovarian tumour cells (Hagemann *et al*, 2005), plays a critical role in CXCR4 expression (Maroni *et al*, 2007) and regulates apoptosis (Beere, 2004). In view of the possible interaction between NF-*κ*B and ETS1 transcription factors on CXCR4 promoter (Maroni *et al*, 2007), we studied ETS1 transactivating activity under our experimental conditions.

We found that in HGF-treated highly invasive MDA-MB231 cells, HDACs and the enhanced/prolonged c-Src phosphorylation were molecular events responsible for the decrease of CXCR4 expression through NF-*κ*B and the reduction of chemoinvasion. Trichostatin A only partly counteracted HGF-inhibitory effect on chemoinvasion because it enhanced CXCR4 expression by ETS1 activation/c-Src activity reduction, but triggered apoptosis through inhibition of the Akt/NF-*κ*B pathway. The diminished CXCR4 function at the secondary growth site, where HGF seems to be produced (Matsumoto and Nakamura, 2006; Benvenuti and Comoglio, 2007), probably has biological implications: the homing of metastatic cells such as MDA-MB231, attracted by CXCL12, might be enhanced if HGF reduced their motility.

## Materials and methods

### Materials

Fetal bovine serum (FBS), RPMI-1640, TSA and AMD3100 were from Sigma Chemical Co. (St Louis, MO, USA). Recombinant human HGF and anti-CXCR4 monoclonal antibody (MAB-182) were from R&D Systems (Abingdon, UK). Anti-acetyl-histone H4, anti-histone H4, anti-heterochromatin protein 1*γ* (HP1*γ*) (clone 42s2), anti-phosphotyrosine (clone 4G10), anti-c-Src (clone GD11) and anti-phospho-c-Src(Tyr 416) antibodies were from Upstate Biotechnology (Lake Placid, NY, USA). Alexa Fluor568 secondary antibody and JC-1 kit were from Molecular Probes (Eugene, OR, USA). Anti-phospho-Akt (Ser473) and Anti-phosphoMet (Tyr1349) antibodies were from Cell Signalling (Beverly, MA, USA). Anti-Met (C-12), anti-Akt1/2 (H136), anti-ERK1/2 (K-23), anti-phosphoERK1/2 (E-4), anti-p53 (DO-1), anti-p21 (C-19) and anti-vinculin antibodies were from Santa-Cruz Biotechnology (Santa Cruz, CA, USA). Fugene 6 was from Roche Applied Science (Mannheim, Germany). Lipofectamine 2000 was from Invitrogen (Milan, Italy). pRL-TK (*Renilla* luciferase) was from Promega (Madison, WI, USA). SU6656 (c-Src inhibitor), 1L-6-hydroxymethyl-*chiro*-inositol-2-(*R*)-2-*O*-methyl-3-*O*-octadecylcarbonate (Akt inhibitor) and PD 98059 (MEK inhibitor) were from Calbiochem (Darmstadt, Germany).

### Cell cultures and treatments

Human breast carcinoma cells MCF-7 and MDA-MB231 (European Cell Cultures Collection, Salisbury, UK), routinely maintained in RPMI-1640 medium containing 10% FBS, were starved (0.1% FBS) for 18–24 h before HGF treatment (Matteucci *et al*, 2005). Trichostatin A was prepared as a 1 mg ml^−1^ stock solution in absolute ethanol, and stored at −20°C until use (Matheu *et al*, 2005). Dose–response experiments were carried out with TSA, evaluating the effect on histone H4 acetylation, and 2.5 *μ*M caused strong enhancement while 0.1 *μ*M had no effect on H4 acetylation, as reported (Matheu *et al*, 2005). In addition, 2.5 *μ*M TSA caused dissociation of HP1*γ* from chromatin and loss of heterochromatin (Supplementary Figure 1A and B). Thus, we tested the two TSA doses on the different parameters.

### Transient transfection and luciferase reporter assay

We used the gene reporters pCXCR4(−2632/+86)Luc, p0.38SRCLuc cloned in the pGL2-enhancer vector (Dehm *et al*, 2004; Maroni *et al*, 2007), and NF-*κ*BLuc containing three NF-*κ*B consensus sequences from M Hung (Anderson Cancer Center, Houston, TX, USA). The gene reporter construct p0.38SRCA1-CAT (kindly given by K Bonham, Saskatchewan Cancer Agency, Saskatoon, Canada) was cut with *Sca*I and *Hin*dIII, and the fragment excised was subcloned in PGL2-enhancer vector, previously cut with *Sma*I and *Hin*dIII. The ETS1Luc containing five ETS1 consensus sequences was prepared from p600Luc construct (Maroni *et al*, 2007), by deleting two bases (the third and fourth C) inside the p65 consensus site core (TCCC) with the QuickChange site-directed mutagenesis kit (Stratagene, La Jolla, CA, USA) and the following PCR primers: forward 5′-TCCCCTGGGCTTCAAGCCGCGCACCTCT; reverse 5′-AGAGGTGCGCGGCTTGAAGCCCAGGCGA. The cells, in 24-multiwell plates, were transfected with a DNA/Fugene 6 mixture containing 200 ng of each promoter construct and 40 ng *Renilla* luciferase (for normalisation) per well, treated with TSA and collected 1 or 2 days later. Some cells were exposed concomitantly for 1 day to 2.5 *μ*M TSA and 2 *μ*M c-Src inhibitor (SU6656) (Blake *et al*, 2000) or 1 *μ*g per well dominant negative for c-Src (SrcK295M) or ETS1 (ΔEBHHB, ΔETS1) (kindly given by WC Horne, Yale University, New Haven, CT, USA and J Ghysdael, Institut Curie, Orsay, France). Some TSA-treated cells were transfected with 200 ng per well c-Src wild-type (Srcwt) expression vector from S Parson (University of Virginia, Charlottesville, VA, USA). For 1-day HGF treatment (200 ng ml^−1^), the cells were previously starved overnight with or without 2.5 or 0.1 *μ*M TSA, or 2 *μ*M c-Src inhibitor or 8 *μ*M Akt inhibitor (Hu *et al*, 2000; Cheng *et al*, 2005), or were transfected with c-Src dominant negative. Firefly/*Renilla* luciferase activity ratios were calculated by the software, using the readings obtained with the dual luciferase assay system (Promega). Transfection efficiency was 20–25% for MCF-7 and MDA-MB231 cells, evaluated in *β*-galactosidase-stained cells (Matteucci *et al*, 2007).

### Transfection of small interfering RNA (siRNA)

Transfection of siRNA (200 pmol ml^−1^) targeting against Met was carried out according to the manufacturer's instructions (Dharmacon Inc., Lafayette, CO, USA) using lipofectamine 2000 (Song *et al*, 2007). As a control, to assess the specificity of the effects, we used antiluciferase siRNA. Two days after transfection, with or without TSA during the last day, the cells were used for total protein extraction with urea lysis buffer, followed by western blot analysis.

### Tumour cell chemoinvasion assay

Matrigel invasion chambers from BD Biocoat Cellware (Becton Dickinson Labware, Bedford, MA, USA) were used. Starved cells (8 × 10^4^ per well), either untreated or pretreated for 1 day with HGF (200 ng ml^−1^) with or without 2.5 *μ*M TSA, AMD3100 (1 *μ*g ml^−1^) (Uchida *et al*, 2007) or protein kinase inhibitors, were seeded in the top chamber and CXCL12 (200 ng ml^−1^ of medium without serum) was added to the bottom chamber. After 22 h incubation in a humidified tissue culture incubator, non-invading cells were removed from the top, and invading cells were stained with Diff-Quick (Dade Bering, Switzerland). Ten fields under × 200 magnification were randomly selected and counted (Matteucci *et al*, 2005).

### Fluorescence microscopy

The cells (4 × 10^4^), seeded on sterile coverslips placed in 24-multiwell plates, were starved with or without 2.5 *μ*M TSA, treated with HGF (200 ng ml^−1^), fixed with 4% paraformaldehyde solution and permeabilised with 0.2% Triton X-100. They were then incubated with anti-HP1*γ* antibody (1 : 1000) for 2 h, followed by reaction with Alexa Fluor568 secondary antibody (1 : 800), and nuclear staining with DAPI (1 : 2000). Using a fluorescence microscope (Leica TCS SP2-A0BS), the images were collected at × 400 magnification and displayed on a computer screen. Ten fields under × 200 magnification were randomly selected and counted (Matteucci *et al*, 2005).

### Western blot assays and immunoprecipitation experiments

Starved cells were treated with 2.5 or 0.1 *μ*M TSA with or without HGF (200 ng ml^−1^) for 1 or 2 days. Some cells were treated for 1 day with c-Src or Akt inhibitor. Total proteins or nuclear proteins, enriched in histones, were extracted. Western blots of total proteins (100 *μ*g) and nuclear proteins containing histones (5 *μ*g) were carried out with respectively 10–12 and 20% SDS–polyacrylamide gels (Yoshida *et al*, 1990; Nair *et al*, 2004; Matteucci *et al*, 2007). Immunoblots were carried out with anti-acetyl-histone H4 (2 *μ*g ml^−1^), anti-histone H4 (0.5 *μ*g ml^−1^), anti-CXCR4 (5 *μ*g ml^−1^), anti-Met (1 : 200), anti-phosphoMet (1 : 1000), anti-caspase 3 (1 : 1000), anti-caspase 8 (1 : 1000), anti-Bax (1 : 500), anti-Akt1/2 (1 *μ*g ml^−1^), anti-phosphoAkt1/2 (1 : 1000), anti-ERK1/2 (1 : 200), anti-phosphoERK1/2 (1 : 200), anti-c-Src (1 *μ*g ml^−1^), anti-phospho-c-Src (2 *μ*g ml^−1^), anti-p53 (1 : 1000) or anti-p21 (1 : 1000). To confirm equal loading of total and nuclear proteins, the membranes were immunoblotted respectively with anti-vinculin and anti-histone H4 antibodies (Nair *et al*, 2004; Matteucci *et al*, 2007). Total protein extracts (1 mg of protein) were immunoprecipitated with 2 *μ*g of anti-Met antibody. For comparison of the two cell lines, the filters were concomitantly hybridised and manipulated in the subsequent passages. The signals, detected using an enhanced chemiluminescence kit (ECL or ECL-plus; Amersham Biosciences, Amersham, UK), were evaluated by densitometric analysis. Then the relative amounts of proteins were calculated (Matteucci *et al*, 2005).

### JC-1 mitochondrial membrane potential detection and flow cytometry

We used the fluorescent cationic dye 5,5′,6,6′-tetrachloro-1,1′,3,3′-tetra-ethyl-benzamidazolycarbocyanin iodide (JC-1) for *in situ* detection of mitochondrial membrane transition events in live cells, which provides early initiation of cellular apoptosis, according to the manufacturer's instructions. In non-apoptotic cells, JC-1 is in the monomeric form in the cytosol (green) and also accumulates as aggregates in the mitochondria (red). In apoptotic and necrotic cells, JC-1 exists only in the monomeric form and stains the cytosol (green). Starved MCF-7 and MDA-MB231 cells were treated with 2.5 or 0.1 *μ*M TSA for 1 and 2 days. The cells were incubated with JC-1 at 37°C in the dark for 1 h, then analysed with a flow cytometer (Flomax, Partec, Münster, Germany).

### Statistical analysis

Luciferase activity, densitometric values and the number of migrated cells were analysed by analysis of variance, with *P*<0.05 considered significant. Differences from controls were evaluated on original experimental data.

## Results

### HGF had opposite effects on the invasiveness of MDA-MB231 and MCF-7 cells involving HDACs

**Figure 1:**
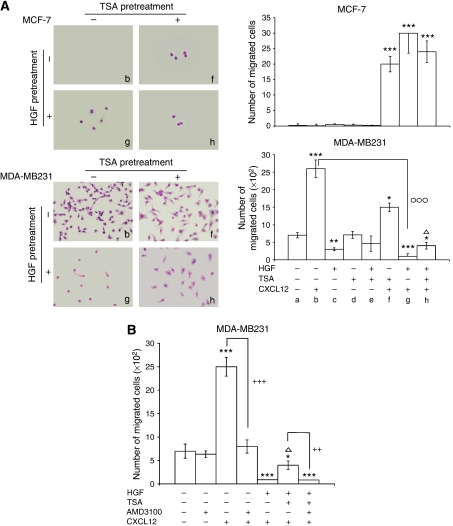
Effect of HGF and role of HDACs in the invasiveness of breast carcinoma cells. (**A**) MCF-7 and MDA-MB231 cells pretreated with HGF (200 ng ml^−1^) for 1 day, with or without 2.5 *μ*M TSA, were used for the Matrigel invasion assay, with CXCL12 (200 ng ml^−1^) in the bottom chamber. To estimate invasion, we counted the invading cells on the lower side of the membrane after staining ( × 200 magnification of selected fields). Representative images, corresponding to columns **b**, **f**, **g** and **h** of the histograms, are shown. The histograms report the number of migrated cells in 10 fields. The experiments were repeated eight times, and the means±s.e. are shown. ^*^P<0.05, ^**^P<0.005 and ^***^P<0.001 *vs* respective starved cells; ^Δ^*P*<0.05 *vs* HGF-pretreated cells; °°°*P*<0.001 *vs* CXCL12 exposed cells. (**B**) Some MCF-7 and MDA-MB231 cells, pretreated with HGF for 1 day, were exposed to 2.5 *μ*M TSA with or without AMD3100 (1 *μ*g ml^−1^). The histograms report the number of migrated cells in 10 fields. The experiments were repeated eight times, and the means±s.e. are shown. ^*^P<0.05 and ^***^P<0.001 *vs* respective starved cells; ^Δ^*P*<0.05 *vs* HGF-pretreated cells; ^++^*P*<0.005 and ^+++^*P*<0.001 for AMD3100-treated *vs* AMD3100-untreated cells.

To see whether HGF affected the invasiveness of highly invasive MDA-MB231 cells differently from low-invasive MCF-7 cells, cell migration experiments were carried out with a Matrigel invasion chamber, which is considered an *in vitro* model system for metastasis (Liang *et al*, 2004; Matteucci *et al*, 2005) (Figure 1A). When indicated, the chemoattractant CXCL12 was added in the bottom chamber to induce CXCR4-positive breast cancer cell to invade through the Matrigel (specific chemoinvasion). Concomitant TSA treatment was carried out to evaluate HDAC involvement in these changes.

In Figure 1A, representative images are reported (corresponding to columns b, f, g, h of the histograms). The main findings referred to MDA-MB231 cells (lower histogram), which migrated spontaneously (7.5 × 10^2^ cells in 10 fields) (column a) and even more (25 × 10^2^) with CXCL12 in the bottom chamber (column b). HGF pretreatment caused a diminution (60%) of spontaneously migrated cells (column c *vs* a), suggesting that this cytokine might affect the mesenchymal (motile) phenotype depending on *β*1-integrin binding to collagen and ECM-degrading enzymes (Wolf *et al*, 2008). In addition, the specific chemoinvasion towards CXCL12 was 96% lower in HGF-pretreated cells (column g *vs* b), consistent with the reduced CXCR4 level, as reported later in this paper. Trichostatin A co-treatment partly prevented the inhibitory effect of HGF (column h *vs* g), indicating that HDACs were involved. The 40% reduction of CXCL12-mediated chemoinvasion after TSA (column f *vs* b), however, might be due to its proapoptotic role and a possible direct effect on motility (Boyault *et al*, 2007).

In contrast, the low-invasive MCF-7 cells did not migrate through the Matrigel-coated filter, either with or without CXCL12 in the bottom chamber (see Figure 1 upper histogram, columns a and b). Pretreatment with HGF, TSA or both increased the responsiveness to CXCL12 (columns f, g and h), and the cells displayed significant chemoinvasion properties: the number of migrated cells in 10 fields ranged from 20 to 30.

In preliminary experiments, we found that CXCL12 induced the migration of breast cancer cells through Matrigel in a dose-dependent manner. The optimal invasive response under our experimental conditions was with 100–200 ng ml^−1^ CXCL12 (not shown). Lower doses (50–100 ng ml^−1^, Fernandis *et al*, 2004) and higher ones (400 ng ml^−1^, Liang *et al*, 2004) are generally used to evaluate MDA-MB231 cell chemoinvasion. Pretreatment of breast carcinoma cells with 100 or 200 ng ml^−1^ HGF caused similar changes in invasiveness (not shown).

In MDA-MB231 cells, AMD3100, a competitive antagonist of CXCL12, did not modify basal invasiveness, but completely prevented CXCL12-mediated chemoinvasion and also hindered the effect of TSA in HGF-treated cells (Figure 1B).

### Mechanistic insights of the changes of invasiveness after HGF

**Figure 2:**
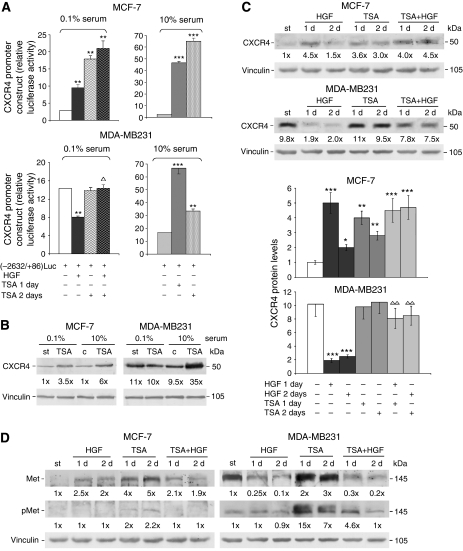
Changes in CXCR4 and Met expression after HGF treatment implicated HDACs. (**A**) The cells were transiently transfected with pCXCR4(−2632/+86)Luc. Some starved cells, treated with 2.5 *μ*M TSA, were added with HGF (200 ng ml^−1^) for 1 day. The other cells in 10% serum were treated with 2.5 *μ*M TSA for 1 or 2 days. The histograms indicate the absolute values for ratios of Firefly/*Renilla* luciferase activity. The data are the means±s.e. of three independent experiments performed in triplicate. ^**^P<0.005 and ^***^P<0.001 *vs* respective control value; ^Δ^*P*<0.05 *vs* HGF-treated MDA-MB231 cells. (**B**) Western blot analyses of CXCR4 protein levels in starved (0.1% serum) and serum-maintained (10% serum) cells treated with 2.5 *μ*M TSA for 1 day. The numbers at the bottom indicate the fold variations relative to the value of starved (st) MCF-7 cells. Immunoblot with anti-vinculin antibody was used for normalisation. The experiments were repeated three times, with similar results. (**C**) Western blot analyses of CXCR4 protein levels in cells treated with HGF with or without 2.5 *μ*M TSA for 1 or 2 days (d). Representative western blots are shown, and the numbers at the bottom indicate the fold variations relative to the value of st MCF-7 cells. Immunoblot with anti-vinculin antibody was used for normalisation. The experiments were repeated five times, with similar results, and the means±s.e. are reported in the histograms. ^*^P<0.05, ^**^P<0.005 and ^***^P<0.001 *vs* respective control values; ^ΔΔ^*P*<0.005 *vs* HGF-treated cells. (**D**) Western blot analyses of Met protein levels in cells treated with HGF with or without 2.5 *μ*M TSA for 1 or 2 days (d). The numbers at the bottom indicate the fold variations relative to the respective st values. Immunoblot with anti-vinculin antibody was used for normalisation. The blots are representative of experiments repeated three times.

To clarify the mechanistic basis of the changes of invasiveness after HGF, and the role of the HDACs, we studied the pattern of CXCR4 transactivation and protein levels and the expression of Met in cells exposed to HGF plus 2.5 *μ*M TSA (Figure 2).

The cells were transiently transfected with the gene reporter driven by the CXCR4 promoter(−2632/+86)Luc, and three treatments were established: TSA in medium containing 10% FBS for 1 or 2 days (i); TSA in medium containing 0.1% FBS for 2 days (ii) and added with HGF during the last day (iii) (Matheu *et al*, 2005; Matteucci *et al*, 2007) (Figure 2A). Some signalling pathways, such as Akt, possibly implicated in CXCR4 expression after HGF treatment, may be influenced by serum (Gort *et al*, 2006).

As shown in Figure 2A, in MDA-MB231 cells HGF reduced the basal luciferase activity of starved cells (by about 40%); the addition of TSA prevented this inhibition. CXCR4Luc activity was not affected by 2-day TSA treatment under starvation, while TSA increased CXCR4 transactivation (2–4-folds) in the presence of 10% FBS. Culture conditions seemed therefore to affect the cell signalling implicated in the regulatory role of HDACs in CXCR4 transactivation. Consistently, only in serum-maintained MDA-MB231 cells, TSA raised CXCR4 protein level (about three-fold) (Figure 2B). To evaluate the involvement of HDACs in HGF effect on CXCR4 protein levels, the cells were starved to prevent interference from serum growth factors (Figure 2C). In MDA-MB231 cells, HGF lowered the CXCR4 protein level (by about 80%); the combination with TSA almost completely prevented this effect, although TSA alone did not affect the CXCR4 protein level, giving values similar to those of starved (st) cells. The experiments were repeated five times, as reported in Figure 2C.

All these findings in MDA-MB231 cells differed from MCF-7 cells, in which HGF alone or with TSA strongly enhanced CXCR4 transactivation, independently of serum (Figure 2A), and raised the CXCR4 protein level (Figure 2B and C). The dose of 0.1 *μ*M TSA did not boost gene reporter activity in either cell line (data not shown).

In conclusion, our experimental conditions also modified carcinoma cell invasiveness by affecting CXCR4. Basal CXCR4Luc activity and protein level were higher in MDA-MB231 than MCF-7 cells. The increases after TSA were, however, bigger in MCF-7 than MDA-MB231 cells, indicating possible differences of HDAC activities and/or their role in the regulation of CXCR4 promoter in the two lines. Possibly in TSA-treated MDA-MB231 cells the activity of signalling pathway(s) and/or transcription factor(s) regulated downstream limit CXCR4 transactivation. Histone deacetylase blockade with TSA in HGF-treated MDA-MB231 cells partly relieved the inhibition of CXCL12-mediated chemoinvasion also by affecting CXCR4. In MCF-7 cells, HGF had stimulatory effects similar to TSA, but invasion through Matrigel under the two stimuli never reached that of MDA-MB231 cells, indicating that CXCR4 receptors were partly functional.

Met protein level was about 20-fold higher in MDA-MB231 than in MCF-7 cells (Figure 2D): in MDA-MB231 cells TSA enhanced (2–3-folds) and HGF reduced (−75 to −90%) the Met protein level. The blockade of HDACs with TSA seemed to induce a functional Met protein phosphorylated at the catalytic domain (Tyr 1349) mostly in MDA-MB231 cells (7–15-folds increase). The mechanism might be linked to receptor overexpression, making HGF ligand binding unnecessary for receptor activation (Benvenuti and Comoglio, 2007): the phosphoMet/Met ratios in TSA-treated and starved cells were 7 and 2.5. Hepatocyte growth factor impaired the increases in Met protein level and phosphorylation after TSA. In MCF-7 cells, HGF or TSA treatment raised the Met protein level (Figure 2D).

### Effects of HGF treatment on signal-transduction pathways possibly implicated in CXCR4-mediated invasion

**Figure 3:**
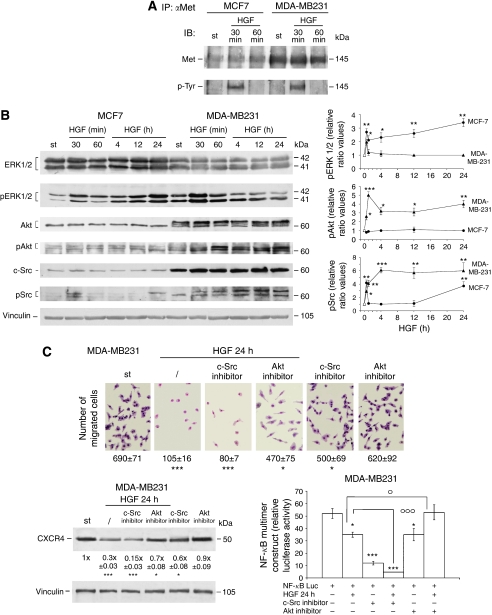
Changes in signalling pathways after HGF. (**A**) Total cell extracts from starved (st) and HGF-treated cells were immunoprecipitated using anti-Met antibody, and immunoblotted with anti-Met and anti-phosphotyrosine (p-Tyr) antibodies. The experiments were repeated three times, with similar results. (**B**) MCF-7 and MDA-MB231 cells were exposed to HGF for various times, and western blots of total proteins were performed in triplicate. Representative western blots are shown. Densitometric analysis was carried out for each cell line, ERK1/2, Akt and c-Src protein levels were not changed by HGF, and were taken as 1. The graphs show phosphoERK1/2/ERK1/2 (pERK1/2), phosphoAkt/Akt (pAkt) and phospho-c-Src/c-Src (pSrc) ratios in starved (○) and HGF-treated (•) MCF-7 cells and in starved (▵) and HGF-treated (▴) MDA-MB231 cells. The data are the means±s.e. of experiments repeated three times. ^*^P<0.05, ^**^P<0.005 and ^***^P<0.001 *vs* respective values for st cells. (**C**) MCF-7 and MDA-MB231 cells pretreated with HGF for 24 h, with or without 2 *μ*M c-Src or 8 *μ*M Akt inhibitor, were used for the Matrigel invasion assay, with CXCL12 in the bottom chamber. We estimated invasion by counting the invading cells on the lower side of the membrane after staining ( × 200 magnification of selected fields). The experiments were repeated three times. The numbers at the bottom indicate the means of migrated cells of 10 fields±s.e. ^*^P<0.05 and ^***^P<0.001 *vs* st cells. Western blot analysis of CXCR4 protein levels in 24 h HGF-treated cells, with or without c-Src or Akt inhibitor. The numbers at the bottom indicate the means±s.e. of three experiments. ^*^P<0.05 and ^***^P<0.001 *vs* st cells. Cells transfected with NF-*κ*BLuc were exposed to HGF for 24 h, with or without c-Src or Akt inhibitor. The histograms indicate the absolute values for ratios of Firefly/*Renilla* luciferase activity. The data are the means±s.e. of five independent experiments performed in triplicate. ^*^P<0.05 and ^***^P<0.001 *vs* control value; °*P*<0.05 and °°°*P*<0.001 *vs* HGF-treated cells.

Hepatocyte growth factor causes early direct molecular changes and indirect late effects (Tacchini *et al*, 2000). We hypothesised that the molecular effects of HGF leading to changes in invasiveness and CXCR4 expression started before the Met downregulation observed at 1 or 2 days in MDA-MB231 cells. Therefore, we studied the time courses of the signalling pathways possibly activated by HGF, and their roles in the changes of invasiveness, of CXCR4 protein levels and of NF-*κ*B transactivation (Figure 3). Nuclear factor-*κ*B is one of the main transcription factors in CXCR4 expression (Maroni *et al*, 2007; Matteucci *et al*, 2007).

In MDA-MB231 cells, HGF transiently enhanced Met tyrosine phosphorylation at 30 min, similar to in MCF-7 cells (Figure 3A). However, the phosphorylation patterns of ERK1/2, Akt and c-Src were different in the two cell lines after HGF treatment (Figure 3B). The numbers in the graphs were calculated using the densitometric values of the western blots and taking as 1 the values of ERK1/2, Akt and c-Src protein levels unmodified by HGF.

In HGF-treated MDA-MB231 cells, phosphoAkt/Akt (pAkt) and phospho-c-Src/c-Src (pSrc) ratios increased starting from 30 min up to 24 h. The phosphoERK1/2/ERK1/2 (pERK) ratio showed a short-lasting rise at 30 min (Figure 3B). In MDA-MB231 cells, Akt migrates at approximately 60 kDa as a tightly spaced triplet (Stoica *et al*, 2003; Matteucci *et al*, 2007). We evaluated phosphoAkt using an antibody directed against Ser 473 of the various isoforms. These results suggested that the HGF/Met interaction, causing rapid Met-tyrosine phosphorylation, regulated downstream transduction pathways before Met receptor downregulation. Akt and c-Src phosphorylations were persistently elevated in HGF-treated MDA-MB231 cells, suggesting that these cell signalling pathways might be important to regulate changes of invasiveness and CXCR4 expression. In contrast, in HGF-treated MCF-7 cells the pERK ratio rapidly and persistently increased for up to 24 h, while the pSrc ratio enhancement was biphasic (Figure 3B).

To understand the role of protein kinases in CXCL12-mediated chemoinvasion in HGF-treated MDA-MB231 cells, we analysed the effect of specific inhibitors (Figure 3C). Hepatocyte growth factor reduced chemoinvasion towards CXCL12 (85%), the CXCR4 protein level (70%) and NF-*κ*B transactivation (33%). These inhibitory effects were enhanced by c-Src blockade with SU6656, but prevented by Akt blockade, to different extents. The MEK inhibitor PD 98059 (100 *μ*M) (Matteucci *et al*, 2005) had no effect on HGF-induced changes (data not shown). These findings suggested that c-Src inhibition might be important to decrease CXCL12-mediated chemoinvasion, possibly involving reduced NF-*κ*B-dependent transactivation of CXCR4, and demonstrated that c-Src inhibition had additive effects with HGF, probably because they controlled different molecular mechanisms in MDA-MB231 cell motility. The findings with the chemical inhibitor of c-Src were confirmed using c-Src dominant negative (data not shown).

### Involvement of c-Src and ETS1 in the changes of CXCR4 transactivating activity induced by TSA

**Figure 4:**
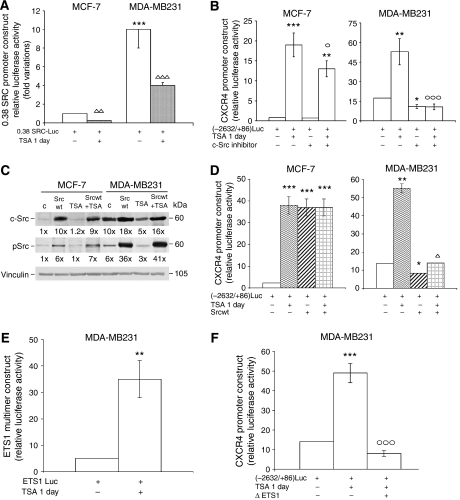
Effects of c-Src and ETS1 on TSA-induced changes of CXCR4 transactivating activity. (**A**) Cells transfected with 0.38SRCLuc were exposed to 2.5 *μ*M TSA in medium containing 10% FBS. The histograms indicate the fold variations of luciferase activity relative to MCF-7 control value, taken as 1. The data are the means±s.e. of five independent experiments performed in triplicate. ^***^P<0.001 *vs* MCF-7 control value; ^ΔΔ^*P*<0.005 and ^ΔΔΔ^*P*<0.001 for TSA treatment *vs* respective values for controls. (**B**) Cells transfected with pCXCR4(−2632/+86)Luc were exposed to 2.5 *μ*M TSA with or without 2 *μ*M c-Src inhibitor in medium containing 10% FBS. The histograms indicate the absolute values for ratios of Firefly/*Renilla* luciferase activity. The data are the means±s.e. of three independent experiments performed in triplicate. ^*^P<0.05, ^**^P<0.005 and ^***^P<0.001 *vs* respective values for controls; °*P*<0.05 and °°°*P*<0.001 *vs* TSA-treated cells. (**C**) Total proteins were extracted 1 day after Srcwt transfection and 2.5 *μ*M TSA treatment, and were analysed by western blot. Immunoblot with anti-c-Src or anti phospho-c-Src (pSrc) antibody was carried out, and the numbers at the bottom indicate the fold variations relative to the MCF-7 control value. Immunoblot with anti-vinculin antibody was used for normalisation. Experiments were repeated three times, with similar results. (**D**) Cells were co-transfected with pCXCR4(−2632/+86)Luc and Srcwt expression vector, and exposed or not to 2.5 *μ*M TSA in medium containing 10% FBS. The histograms indicate the absolute values for ratios of Firefly/*Renilla* luciferase activity. The data are the means±s.e. of three independent experiments performed in triplicate. ^*^P<0.05, ^**^P<0.005 and ^***^P<0.001 *vs* respective control value; ^Δ^*P*<0.05 *vs* Srcwt-transfected MDA-MB231 cells. (**E**) MDA-MB231 cells transfected with ETS1Luc were exposed to 2.5 *μ*M TSA in medium containing 10% FBS. The histograms indicate the absolute values for ratios of Firefly/*Renilla* luciferase activity. The data are the mean±s.e. of five independent experiments performed in triplicate. ^**^P<0.005 *vs* control value. (**F**) Cells co-transfected with pCXCR4(−2632/+86)Luc and ΔETS1 were exposed to 2.5 *μ*M TSA in medium containing 10% FBS. The histograms indicate the absolute values for ratios of Firefly/*Renilla* luciferase activity. The data are the means±s.e. of three independent experiments performed in triplicate. ^***^P<0.001 *vs* control value; °°°*P*<0.001 *vs* TSA-treated cells.

c-Src is a critical component of the pathways regulating proliferation, survival and metastasis (through effects on adhesion, invasion and motility), and is one of the Met-signal transducers involved in the regulation of NF-*κ*B activity (Huang *et al*, 2003a; Yeatman, 2004; Mazzone and Comoglio, 2006). High protein levels and/or catalytic activity of c-Src have been detected in a number of human cancers, including breast carcinoma (Ishizawar and Parsons, 2004). To investigate the functional role of c-Src in TSA-treated cells, we examined endogenous c-Src expression and CXCR4 transactivating activity after c-Src inhibition with SU6656 (Figure 4).

Transfection experiments were carried out with a construct containing the 0.38 kb fragment of the c-Src promoter SCR1A (Figure 4A). The activity of this construct is negatively regulated by HDAC inhibitors due to the presence of core-promoter elements including TAF1 (Dehm *et al*, 2004). 0.38SRCLuc activity was 10 times higher in MDA-MB231 than in MCF-7 cells (the absolute value for Firefly/*Renilla* luciferase activity ratio was 2 × 10^−2^ in MCF-7 cells). In MDA-MB231 cells, TSA treatment reduced the activity of the c-Src promoter construct by 60% (Figure 4A) and of endogenous c-Src protein level by 50% (Figure 4C).

As shown in Figure 4B, the inhibition of basal c-Src activity with SU6656 largely prevented (75%) the CXCR4 transactivating activity of TSA-treated MDA-MB231 cells. The findings with the chemical inhibitor of c-Src were confirmed using c-Src dominant negative (data not shown). These results indicate the important role of endogenous c-Src activity in MDA-MB231 cells for the TSA-stimulatory effect on CXCR4 expression.

To further clarify the role of c-Src, experiments were carried out using the Srcwt expression vector (Figure 4C and D). Similar to HGF treatment, Srcwt permits an evaluation of the effects of enhanced c-Src activity. In fact, the highly expressed exogenous c-Src was phosphorylated, therefore active (Figure 4C).

Thus, in MDA-MB231 cells, Srcwt reduced the basal CXCR4 transactivating activity, possibly by increasing c-Src formation, and interfered with the activation of CXCR4Luc by TSA. In contrast, it was stimulatory on CXCR4Luc in MCF-7 cells (Figure 4D).

To deepen the knowledge of the transcriptional mechanism(s) responsible for the increase in CXCR4 transactivation after TSA treatment in MDA-MB231 cells, we examined ETS1 luciferase activity (Figure 4E and F). Trichostatin A enhanced ETS1 multimer construct activity in MDA-MB231 cells (seven-fold), indicating a key role of this transcription factor (Figure 4E). The ETS1 dominant negative completely prevented CXCR4(−2632/+86)Luc activation by TSA in MDA-MB231 cells (Figure 4F).

### TSA modulated signal-transduction pathways and genes for apoptosis in MDA-MB231 cells

**Figure 5:**
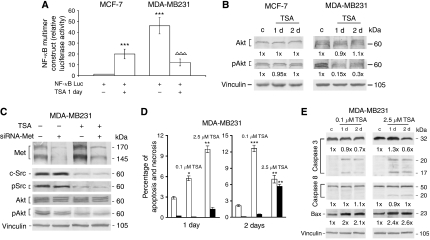
Proapoptotic role of TSA in MDA-MB231 cells. (**A**) Cells transfected with NF-*κ*BLuc were exposed to 2.5 *μ*M TSA in medium containing 10% FBS. The histograms indicate the absolute values for ratios of Firefly/*Renilla* luciferase activity. The data are the means±s.e. of five independent experiments performed in triplicate. ^***^P<0.001 *vs* MCF-7 control value; ^ΔΔΔ^*P*<0.001 *vs* MDA-MB231 control value. (**B**) The cells were exposed to 2.5 *μ*M TSA for 1 or 2 days (d), and western blots of total proteins were carried out. The numbers at the bottom indicate the fold variations relative to the respective values for controls. Immunoblot with anti-vinculin antibody was used for normalisation. The blots are representative of experiments repeated three times. (**C**) TSA-treated cells were transfected with siRNA for Met, and western blots of total proteins were carried out. Anti-Met antibody revealed both the precursor (170 kDa) and the *β*-chain (145 kDa). Immunoblot with anti-vinculin antibody was used for normalisation. The blots are representative of experiments repeated three times. (**D**) The histograms show the percentages of apoptotic and necrotic cells obtained by cytofluorimetric analysis of TSA-treated cells. □, percentage of apoptotic cells; ▪, percentage of necrotic cells. The data are the mean±s.e. of three independent experiments. ^*^P<0.05, ^**^P<0.005 and ^***^P<0.001 *vs* control values at 1 and 2 days. (**E**) The cells were treated with TSA, and total protein extracts were prepared and used for western blots. The numbers at the bottom indicate the fold variations relative to controls. Immunoblot with anti-vinculin antibody was used for normalisation. The blots are representative of experiments repeated three times.

To obtain more information on transcription factors in TSA effects, we studied NF-*κ*B transactivating activity. Nuclear factor-*κ*B seems important for CXCR4 expression (Maroni *et al*, 2007; Matteucci *et al*, 2007) and, depending on cell conditions, it can regulate the triggering of apoptosis (Beere, 2004; Luo *et al*, 2005). Therefore, we examined TSA effect on cell signalling and genes implicated in apoptosis (Figure 5).

In MDA-MB231 cells, 2.5 *μ*M TSA reduced NF-*κ*B transactivation by about 80% (Figure 5A) and the level of phosphorylated (active) Akt (Figure 5B). Opposite effects were seen in MCF-7 cells (Figure 5A and B).

To investigate how Met might be involved in the changes of protein-kinase transducers after TSA, we knocked down the receptor by siRNA-Met in MDA-MB231 cells (Figure 5C). This abolished Met protein enhancement after 2.5 *μ*M TSA for 1 day, but did not prevent the changes of c-Src and Akt and their phosphorylated forms. Antiluciferase-siRNA transfection did not affect the Met control value (not shown).

We verified whether TSA induced apoptosis in MDA-MB231 cells under our experimental conditions (Figure 5D). Cytofluorimetric analysis and JC-1 assay showed that MDA-MB231 cells had a background level of apoptosis of about 2.5%, in agreement with the literature (Toillon *et al*, 2002). Treatment with 0.1 and 2.5 *μ*M TSA for 1 or 2 days raised the percentage of apoptosis (about 6–10% on day 1, and 7–12% on day 2). Necrotic cell death (about 5%) was also observed on day 2 with the higher dose of TSA. Neither TSA dose was apoptotic in MCF-7 cells, and HGF did not give any hallmarks of apoptosis in either cell line (not shown). Dot plots for cytofluorimetric analysis of MCF-7 and MDA-MB231 cells are reported in Supplementary Figure 1C.

To further characterise the TSA-induced apoptotic response, which seemed to be limited to the invasive MDA-MB231 cells, we examined the expression of proapoptotic genes (Figure 5E). These were caspase 3, highly expressed in MDA-MB231 cells, caspase 8, prevalent in MCF-7 cells, and Bax, which is activated by TSA (Huang *et al*, 2003b; Singh *et al*, 2005; Yang *et al*, 2007). In MDA-MB231 cells, procaspase 3 (32 kDa) and 8 (50 kDa) protein levels were practically unchanged, except that procaspase 3 slightly declined 2 days after both TSA doses. Correlated with apoptosis at 1 and 2 days, we observed 17 and 20 kDa cleavage fragments for caspase 3, using 0.1 and 2.5 *μ*M TSA, and 20 kDa cleavage fragment for caspase 8, using 2.5 *μ*M TSA. Bax protein expression was enhanced (2–2.6-folds) after both TSA doses. As expected, the proapoptotic genes were not affected by TSA in MCF-7 cells (not shown).

## Discussion

The main points that call for discussion regard the highly invasive/metastatic MDA-MB231 cells and consist in the HGF-dependent reduction of invasion mediated by CXCL12 due to reduction of CXCR4 levels and function. Interestingly, HDACs played a regulatory role in the reduction of CXCR4 expression that involved critical mechanisms such as enhancement of c-Src and decrease of NF-*κ*B activities. It is worth noting that the blockade of HDACs with TSA induced CXCR4 and Met genes for invasiveness, but also triggered apoptosis through inactivation of the phosphoAkt/NF-*κ*B pathway, partly reducing the number of migrated MDA-MB231 cells. Metastatic cancer cells acquire a motile phenotype to penetrate tissues and vasculature, and adopt survival mechanisms to avoid apoptosis (Mehlen and Puisieux, 2006). Histone deacetylase blockade seemed to modify both these aspects of the aggressive phenotype (Boyault *et al*, 2007). In MCF-7 low-invasive cells, HGF typically enhanced chemoinvasion towards CXCL12, and we found no hallmarks of apoptosis after HDAC blockade.

First, MDA-MB231 cells showed spontaneous mesenchymal movement, related to the key function of proteolytic enzymes (Guo *et al*, 2002; Wolf *et al*, 2008), that decreased after HGF exposure. One explanation might be that HGF reduces ETS1 activity, possibly affecting the expression of target genes such as proteolytic enzymes (Guo *et al*, 2002; Maroni *et al*, 2007; Furlan *et al*, 2008). In addition, HGF largely impaired the striking migration towards CXCL12, that is, specific chemoinvasion, as a consequence of the diminished CXCR4 expression. In the presence of CXCL12, both the inhibitory effects of HGF might be evident.

The early changes in Met phosphorylation after HGF and the persistent activation of transduction-signalling pathways downstream, including c-Src and Akt phosphorylation, seemed to be involved in the later decrease of CXCR4 expression. We have previously suggested that in HGF-treated MDA-MB231 cells phospho-c-Src activates HDAC3 (Matteucci *et al*, 2007), and phosphoAkt stabilizes HDAC3 (Zeng *et al*, 2006). As a corepressor of NF-*κ*B (Chen *et al*, 2001; Gao *et al*, 2005), HDAC3 probably intervenes in the inhibitory regulation of CXCR4 promoter activity. Nuclear factor-*κ*B is one of the principal transcription factors in CXCR4 expression (Maroni *et al*, 2007; Matteucci *et al*, 2007).

We showed that HDACs might really be involved in the HGF-dependent decrease of CXCR4 expression and of chemoinvasion towards CXCL12. Trichostatin A, a competitive inhibitor of HDACs, did in fact reduce the inhibitory effect of HGF on CXCR4 expression and invasiveness. The experimental evidence using a c-Src inhibitor showed that the depletion of endogenous c-Src activity cooperated with HGF to reduce MDA-MB231 cell invasiveness/CXCR4 expression/NF-*κ*B transactivation. Our findings suggest that different molecular mechanisms controlled by c-Src, both after HGF activation and under basal conditions, intervened in NF-*κ*B activity regulation. c-Src maintains NF-*κ*B activity through tyrosine phosphorylation of I-*k*B kinase *α*/*β* (Huang *et al*, 2003a), besides activating HDAC3 (Matteucci *et al*, 2007).

In contrast with c-Src inhibition, the Akt inhibitor hindered HGF inhibitory effects, indicating that Akt activity participated in HGF-reduction of MDA-MB231 cell invasiveness regulating CXCR4 expression through NF-*κ*B (deGraffenried *et al*, 2004).

These findings thus point to the HGF–HDAC interaction as a further mechanism by which the microenvironment influences the tumour invasive/metastatic phenotype at epigenetic level (Matteucci *et al*, 2007; Matsumoto *et al*, 2008). The HGF-dependent decrease in CXCR4 function in MDA-MB231 cells seemed to support our idea that HGF, possibly produced at secondary growth site(s), might enhance the homing of metastatic cells attracted by CXCL12 to specific districts such as bones and lymph nodes (Balkwill, 2004). The site of metastasis depends on the characteristics of neoplastic cells and of the permissive microenvironment in the metastatic target organ for incoming cancer cells (Yilmaz *et al*, 2007). Chemokines and growth factors, such as HGF, seem to act as mediators between the tumour microenvironment and the neoplastic cells, and are important in tumour progression and metastasis (Matsumoto and Nakamura, 2006; Benvenuti and Comoglio, 2007; Desiderio, 2007; Singh *et al*, 2007).

Second, we examined the mechanistic basis of the differences in breast tumour cell responses to TSA, depending on tumour aggressiveness, and considered the transcriptional regulation of CXCR4 and Met, two genes important for tumour cell invasiveness (Benvenuti and Comoglio, 2007; Furlan *et al*, 2008). The growing list of non-histone proteins regulated by acetylation/deacetylation comprises more than 30 transcription factors including NF-*κ*B and ETS1 (Pazin and Kadonaga, 1997; Gao *et al*, 2005; Ducasse and Brown, 2006; Ropero and Esteller, 2007; Wang *et al*, 2007). Six ETS1 and one NF-*κ*B-binding sites are present in the CXCR4 promoter (Maroni *et al*, 2007), and their contribution to CXCR4 expression might depend on different transcription factor activities in MDA-MB231 and MCF-7 cells. Nuclear factor-*κ*B activity is regulated by post-translational modifications, and HDAC inhibitors might activate NF-*κ*B by maintaining high levels of acetylation (Mayo *et al*, 2003), as probably occurred in MCF-7 cells. At a difference, in MDA-MB231 cells with a constitutively active NF-*κ*B (Matteucci *et al*, 2007), p52 subunit acetylation after TSA may well prevent nuclear p65 DNA binding (Hu and Colburn, 2005). Studies in progress in our laboratory show that p52 is acetylated in MDA-MB231 cells transfected with siRNAs for HDACs 1 and 3. The inhibition of NF-*κ*B activity seemed to explain the small enhancement of CXCR4 transactivation after TSA in MDA-MB231 cells, although serum culture-conditions also influence this. Other transcription factors such as ETS1 might be activated by HDAC blockade, regulating CXCR4 transactivation in MDA-MB231 cells. Sites enriched in acetylated histone 3 act as enhancer regulating ETS1 sequences in uPA promoter (Wang *et al*, 2007).

Interconnected molecular events orchestrated by ETS1 in breast tumour cells converge to promote invasion of their matrix environment. The ETS1 appears to favour HGF signalling by triggering HGF activation from the precursor and its receptor Met overexpression, due to the presence of numerous (four) ETS1 sequences (Benvenuti and Comoglio, 2007; Furlan *et al*, 2008). The ETS1 overexpression promotes malignancy through HGF/Met-mediated motility, which allows breast cancer cells to bypass the requirement of promigratory signals supplied by the environment (such as fibronectin) for invading (Furlan *et al*, 2008).

Thus, we suggest that HDAC inhibitors are involved indirectly in Met and CXCR4 induction by controlling the activities of transcription factors with different mechanisms that possibly depended on carcinoma cell aggressiveness. They included NF-*κ*B subunit acetylation, which is currently being studied, and/or ETS1/NF-*κ*B cooperation (Maroni *et al*, 2007). The demonstration of CXCR4 transactivation using a promoter construct, which is likely to have altered nucleosome structures, would exclude the involvement of acetylated histones H3 and H4 after TSA treatment.

Third, our data indicate that the marked decrease of c-Src expression was a key event for TSA effect on the cellular and molecular parameters, including the decrease in the pAkt/Akt ratio in MDA-MB231 cells. The decrease in c-Src expression after HDAC inhibition is more likely direct and results from acetylation of non-histone factors associated with the c-Src promoter (Dehm *et al*, 2004). Reduced c-Src activity is involved in PTEN activation and Akt activity inhibition (Lu *et al*, 2003; Pan *et al*, 2007). However, some effects such as Akt dephosphorylation might also be facilitated by TSA, which alters the dynamics of HDAC-protein phosphatase 1 (Chen *et al*, 2005).

The reductions of c-Src expression, pAkt/Akt ratio and NF-*κ*B activity in TSA-treated MDA-MB231 cells probably contributed to the apoptotic switch, and to the drop in the number of migrated cells. We cannot exclude that TSA might directly affect the function of cytoskeleton proteins and motility (Boyault *et al*, 2007). Only in MDA-MB231 cells did the two doses of TSA (0.1 and 2.5 *μ*M) cause a small percentage of apoptosis, consistent with activation of proapoptotic genes. There are conflicting data on the role of ETS1 in apoptosis (Dittmer, 2003).

Finally, some HGF/Met transduction signals seemed to compete with that triggered by TSA, with opposite effects on chemoinvasion and CXCR4 expression in MDA-MB231 cells. These quite likely consisted in the HGF-dependent enhancement of c-Src phosphorylation, which counteracted the reductions of c-Src transactivation and protein level due to TSA. Srcwt transfection, by increasing active c-Src, reduced the stimulatory effect of TSA on CXCR4 transactivation. Hepatocyte growth factor and TSA had opposite effects on Akt phosphorylation, which seems to have an important influence on the pro- and antiapoptotic role of NF-*κ*B (Mayo *et al*, 2003; Beere, 2004; Luo *et al*, 2005).

In conclusion, HDACs participated in the molecular events activated by HGF, leading to reduction of specific invasiveness in MDA-MB231 cells. Clearly, therefore, the potential therapeutic use of TSA should take into account the differences in the aggressiveness of breast carcinoma cells and the tumour microenvironment at the secondary growth site, where HGF might contribute to creating a permissive niche for homing.
