# Supplementary figures and images for: Inhibitory effect of HGF on invasiveness of aggressive MDA-MB231 breast carcinoma cells, and role of HDACs
Source: Br J Cancer. 2008 Oct 21;99(10):1623–34. doi: 10.1038/sj.bjc.6604726 (PMC2584948; doi:10.1038/sj.bjc.6604726)

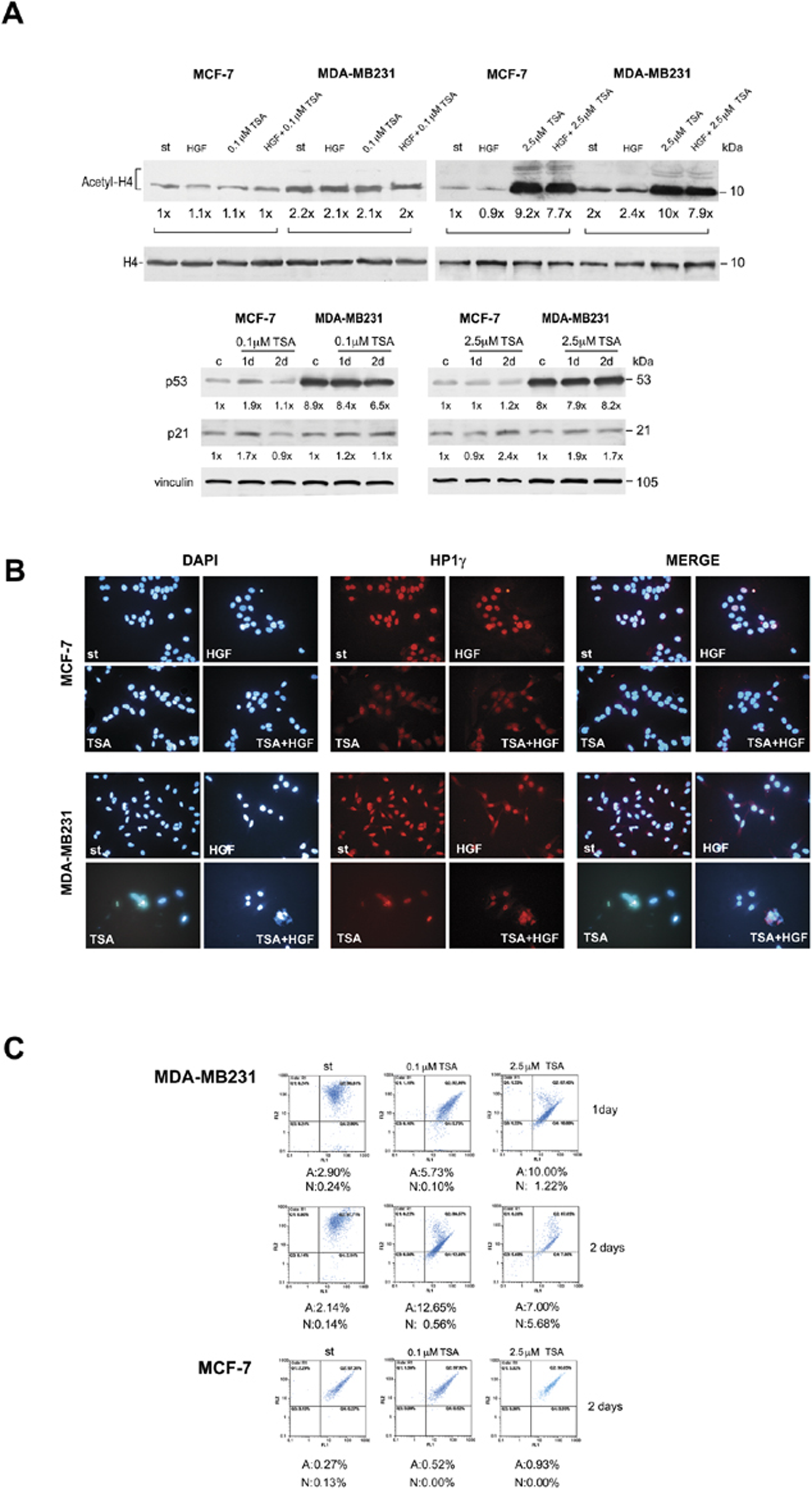

Supplement: Supplementary Figure 1 [file 6604726x1.tif]
