# Supplementary material for: Inhibitory effect of HGF on invasiveness of aggressive MDA-MB231 breast carcinoma cells, and role of HDACs
Source: Br J Cancer. 2008 Oct 21;99(10):1623–34. doi: 10.1038/sj.bjc.6604726 (PMC2584948; doi:10.1038/sj.bjc.6604726)
Supplement: Supplementary Material [file 6604726x2.doc]

# SUPPLEMENTARY MATERIAL

**(A)**

Effects of TSA on acetyl-H4, p53 and p21. HGF was added during the last day of the two-day TSA treatment. Nuclear and total proteins were extracted, examined by Western blot and immunoblotted with anti-acetyl-H4 (nuclear proteins) or anti-p53 and anti-p21 (total proteins). Anti-H4 and anti-vinculin antibodies were used for normalization. The values at the bottom indicate the fold-variations relative to the MCF-7 control value. These experiments were designed to evaluate the efficacy of the TSA treatment, which did indeed increase H4-acetylation at the 2.5 µM dose. TSA probably stabilized p53 in MCF-7 cells, and in MDA-MB231 cells there was depletion after the low dose, probably because of restoration of p53 function. MDA-MB231 cells are mutated at amino acid 280 of p53 (Blagosklonny *et al,* 2005). p21 protein induction might depend on transcriptional activation. The blots are representative of experiments repeated three times.

**(B)**

Immunofluorescence of heterochromatin protein 1γ (HP1γ)-associated chromatin in cells treated with 2.5 µM TSA for two days with or without HGF on the second day. The experiments were performed in triplicate. The literature reports that blockade of histone deacetylation by HDAC inhibitors results in dissociation of HP1 proteins from chromatin and loss of heterochromatin. In MCF-7 cells, TSA with or without HGF reduced the specific fluorescent signal corresponding to HP1γ in practically all the cells examined, compared to starved cells (st). However, after HGF alone HP1γ remained chromatin-associated, as in st cells; this was evidenced by the strong red signal and by the merge image. In MDA-MB231 cells TSA treatment, with or without HGF, caused apoptotic hallmarks in some nuclei (5-10%), while in the others the HP1γ fluorescent signal was reduced. HGF maintained heterochromatin in MDA-MB231 cells too.

**(C)**

Cytofluorimetric analysis of cells treated with 0.1 and 2.5 µM TSA, using JC-1 assay. The experiments were performed in triplicate, with similar results.
